# Machine Learning-Guided Stress Atlases Reveal Co-Expression Rewiring and Divergent Cellular Deployment of Abiotic Stress Programs in Rice and Wheat

**DOI:** 10.3390/ijms27157005

**Published:** 2026-08-04

**Authors:** Zixuan Wang, Zhouxuan Ge, Haoyu Chao, Alibek Zatybekov, Qirong He, Xiaoying Zheng, Yue Wang, Shilong Zhang, Zhimeng Zhao, Renbo Yao, Vladimir A. Ivanisenko, Cong Feng, Ming Chen

**Affiliations:** 1 Department of Bioinformatics, College of Life Sciences, Zhejiang University, Hangzhou 310058, China; zixuan.wang@zju.edu.cn (Z.W.);; 2 Laboratory of Molecular Genetics, Institute of Plant Biology and Biotechnology, Almaty 050040, Kazakhstan; 3 College of Information Science and Electronic Engineering, Zhejiang University, Hangzhou 310058, China; 4 Laboratory of Fruit Quality Biology, The State Agriculture Ministry Laboratory of Horticultural Plant Growth, Development and Quality Improvement, Fruit Science Institute, College of Agriculture and Biotechnology, Zhejiang University, Hangzhou 310058, China; 5 Zhejiang University–University of Edinburgh Institute, Zhejiang University School of Medicine, Zhejiang University, Haining 314400, China; 6 Institute of Cytology and Genetics, Siberian Branch of Russian Academy of Sciences, Novosibirsk 630090, Russia; 7 State Key Laboratory of Vegetation Structure, Function and Construction, College of Life Sciences, Zhejiang University, Hangzhou 310058, China; 8 Zhejiang Key Laboratory of Multi-omics Precision Diagnosis and Treatment of Liver Diseases, Zhejiang University, Hangzhou 310058, China

**Keywords:** abiotic stress, co-expression network, machine learning, rice, single-cell transcriptomics, stress-responsive genes, transcriptome atlas, wheat

## Abstract

Abiotic stress limits cereal productivity, yet whether conserved stress-responsive genes retain similar transcriptional network organization and cellular deployment across cereal species remains unknown. Here, we developed a machine learning-guided comparative framework to integrate public leaf transcriptomes of rice (*Oryza sativa*) and wheat (*Triticum aestivum*) under major abiotic perturbations. Using harmonized compendia comprising 787 rice and 337 wheat samples, we found that rice samples resolved into more discrete stress-associated transcriptional states, whereas wheat samples formed a more continuous landscape with partial overlap among stress responses. Supervised learning prioritized compact sets of stress-predictive candidate genes, including heat-shock/chaperone-related, ABA-biosynthetic and membrane-associated features. Orthology-guided co-expression analysis further showed that homologous stress-predictive genes, particularly heat-associated candidates, can retain stress responsiveness while occupying divergent module neighborhoods in rice and wheat. Leaf single-cell projection resolved this divergence at cellular resolution: several rice predictors showed compartmentalized expression in parenchyma and vascular parenchyma, whereas their wheat counterparts were more broadly deployed across mesophyll, epidermal and vascular cell types. Our findings reveal context-dependent redeployment of homologous stress-associated genes in cereals and offer a scalable strategy for prioritizing candidates for functional validation and climate-resilience breeding.

## 1. Introduction

Rice (*Oryza sativa*) and wheat (*Triticum aestivum*) are two major cereal crops that directly underpin global food security [1,2]. Their productivity is increasingly threatened by environmental extremes, including heat, drought and cold stress, which destabilize yields across diverse agroecosystems [3,4,5]. Plant responses to abiotic stress involve multiple conserved molecular layers, including stress perception, osmotic and hormonal signaling, redox regulation, transcriptional reprogramming, protein homeostasis and metabolic adjustment [6,7]. However, rice and bread wheat differ sharply in genome architecture: rice is a diploid species with a relatively compact reference genome [8], whereas bread wheat is a hexaploid allopolyploid with a large and highly redundant genome [9,10]. These contrasts provide a powerful comparative system for asking how stress-responsive transcriptional programs are conserved, redistributed or specialized across cereal lineages.

Large-scale transcriptomic studies have substantially advanced our understanding of cereal stress responses. In rice, Wilkins et al. reconstructed environmental gene regulatory influence networks in leaves exposed to water deficit, high temperature and agricultural environments, demonstrating that stress-responsive expression programs can be organized at the network level [11]. Cohen and Leach further used public rice transcriptomes to define a core stress response shared by abiotic and biotic perturbations [12]. Rice meta-analyses have identified drought- or multi-stress-responsive gene sets from public transcriptomic resources [13,14]. In wheat, the polyploid transcriptional landscape described by Ramírez-González et al. provided a foundation for understanding homoeolog expression and tissue-level transcriptional organization [10], while recent multi-omics and transcriptomic meta-analyses have characterized wheat responses to single and combined abiotic stresses [15,16]. Integrative omics studies have also begun to connect gene expression, genetic association and functional validation for wheat drought-related traits, as illustrated by the identification and validation of *TaMYB7-A1* as a regulator of water-use efficiency and drought resilience [17]. Together, these studies show the power of large-scale transcriptomics and integrative omics for identifying stress-responsive genes and pathways.

Despite these advances, two related gaps remain. First, most cereal stress studies remain species-centered, making it difficult to determine whether homologous genes in rice and wheat occupy comparable transcriptional contexts. Second, many comparative analyses still emphasize differential expression or sequence orthology, which can identify conserved stress-responsive genes but does not resolve whether those genes are embedded in similar co-expression neighborhoods. This distinction is especially important in comparisons between diploid rice and hexaploid wheat, where gene redundancy, homoeolog expression and stress-associated module organization may alter how homologous genes are deployed within the broader transcriptomic network.

Machine learning is particularly well suited for comparative transcriptomic studies because abiotic stress responses arise from coordinated changes across thousands of highly correlated genes rather than isolated transcriptional events. Conventional differential-expression analyses are highly effective for identifying individual responsive genes but are not specifically designed to prioritize compact multivariate predictive signatures from heterogeneous transcriptomic collections. Supervised machine-learning algorithms can exploit complex multivariate expression patterns, including nonlinear relationships among genes, to identify biologically informative predictors while reducing feature redundancy [18]. When integrated with co-expression network analysis, machine learning further provides a mechanistic framework in which highly predictive genes can be interpreted within their transcriptional neighborhoods rather than as isolated markers. Such an integrative strategy is particularly advantageous for cross-species analyses because differences in genome architecture and regulatory organization may mask conserved stress-associated functions when genes are evaluated individually.

Recent computational studies provide a methodological basis for moving beyond single-gene comparisons. Co-expression network analysis can organize stress-responsive genes into coordinated modules [19]. Machine-learning applications are increasingly used in genomics [18], and network-based machine learning has been applied to plant stress transcriptomes [20]. In rice, network-based machine learning has been used to prioritize transcription factors involved in drought resistance by integrating gene regulatory network connectivity with supervised prediction [20]. Machine learning has also been used to predict rice transcriptional responses to heat and drought from genomic features and to interpret stress-specific feature importance [21]. More broadly, machine-learning meta-analysis has recently been applied to heterogeneous public transcriptomic datasets to extract abiotic-stress gene cores and evaluate their biological robustness [22,23]. However, these approaches have rarely been extended to a cross-species cereal framework that links predictive stress markers, orthologous relationships and co-expression topology.

Recent single-cell resources now make it possible to add a cellular layer to comparative stress transcriptomics [24]. Rice single-cell atlases have resolved major leaf and root cell types and revealed cell-type-specific transcriptomic programs [25,26], and a rice single-cell multi-omics atlas further connected cell-type-resolved expression, chromatin accessibility and regulatory networks across multiple organs [27]. For wheat, leaf single-cell transcriptomic analysis under PEG-induced stress has provided a reference for annotating major wheat leaf cell types and studying drought-related cellular heterogeneity [28]. These resources create an opportunity to ask whether bulk-derived stress-predictive genes are broadly expressed across leaf tissues or preferentially associated with specific cellular compartments. This distinction is important because apparent divergence in bulk stress responses may reflect not only differences in gene-level responsiveness but also differences in cell-type-associated deployment.

Despite extensive transcriptomic characterization of abiotic stress responses in individual cereal species, it remains unclear whether homologous stress-responsive genes in crops with contrasting genome architectures are embedded in comparable transcriptional networks and cellular compartments. Resolving this question is essential for understanding how conserved stress-associated functions are maintained, rewired, or redistributed during cereal evolution. Here, we established a machine learning-guided comparative transcriptomic framework to examine the conservation and divergence of abiotic stress programs between rice and wheat. By integrating harmonized leaf RNA-seq compendia with marker-gene detection, co-expression network analysis, supervised classification, orthology-guided comparison and cell-type-resolved transcriptomic resources, we investigated how stress-associated genes are organized and redeployed across network and cellular contexts in these two major cereal crops.

## 2. Results

### 2.1. Integrated Leaf Transcriptomic Atlases Define Stress-Associated Transcriptional States

To define global transcriptional landscapes associated with abiotic perturbations, we integrated curated public leaf RNA-seq datasets from rice and wheat representing major abiotic stress conditions with sufficient sample representation. Sample metadata, stress annotations and project information are summarized separately for rice and wheat in Supplementary Tables S1 and S2, respectively. After normalization and batch correction within each species, the harmonized expression matrices were embedded using UMAP to visualize major axes of transcriptional variation across samples.

**Figure 1:**
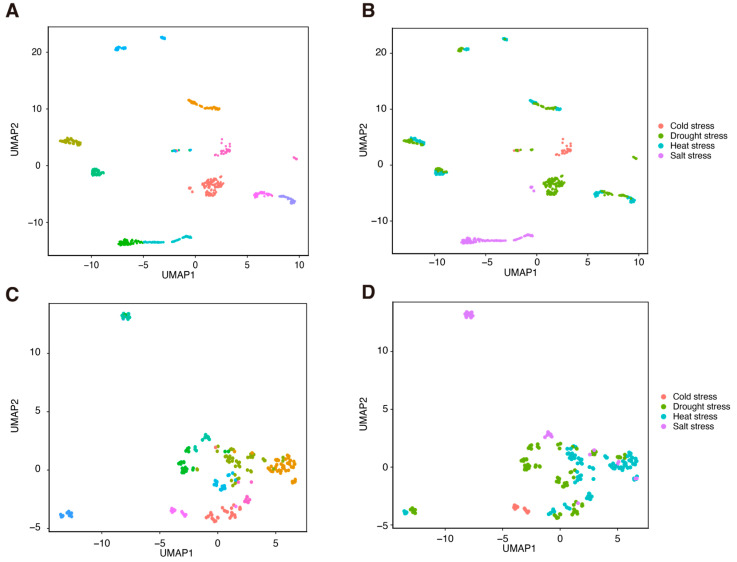
Integrated transcriptomic landscape of cereal leaf samples under abiotic stress. (**A**) UMAP projection of harmonized rice leaf transcriptomes (*n* = 787), colored by cluster identity; (**B**) UMAP projection shown in (**A**), colored by abiotic stress condition; (**C**) UMAP projection of harmonized wheat leaf transcriptomes (*n* = 337), colored by cluster identity; (**D**) UMAP projection shown in (**C**), colored by abiotic stress condition.

In rice, the integrated atlas resolved ten transcriptional clusters, indicating discrete expression states associated with major stress conditions (Figure 1A). Projection of stress labels onto the UMAP manifold revealed clear stress-associated organization of the rice transcriptome (Figure 1B). Several clusters were enriched for specific stress labels, including cold- and salt-associated clusters, whereas other clusters contained mixed labels, suggesting partial overlap among transcriptional programs. 

In wheat, a similar analysis identified nine transcriptional clusters (Figure 1C). Compared with rice, the wheat landscape appeared more continuous, with partial overlap among stress-associated samples, particularly between drought- and heat-related responses, although several clusters still showed preferential enrichment for individual stress labels (Figure 1D). This pattern indicates that the harmonized wheat compendium captured a less discretely partitioned stress-associated expression landscape than the rice compendium.

To capture the transcriptional features underlying these cluster-defined states, we identified cluster-specific marker genes for each species, yielding 3457 marker genes in rice and 4171 marker genes in wheat. These marker-gene sets were subsequently used as a stress-informative feature layer for WGCNA-based co-expression analysis and machine-learning-based candidate prioritization. Detailed marker-gene statistics and cluster assignments are provided separately for rice and wheat in Supplementary Tables S3 and S4, respectively.

### 2.2. Co-Expression Network Analysis Organizes Stress-Informative Genes into Species-Specific Modules

**Figure 2:**
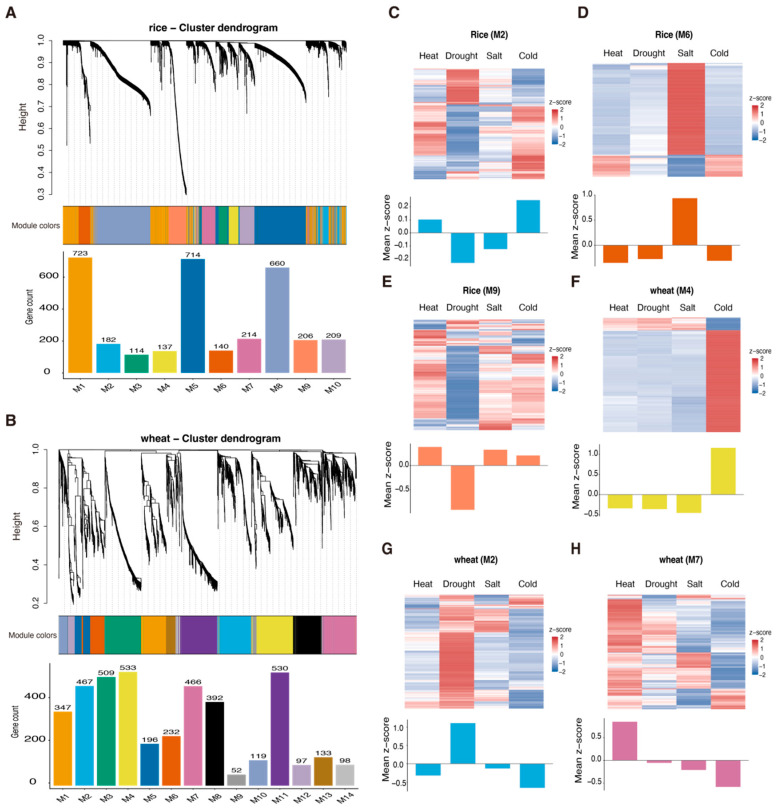
WGCNA identifies stress-associated co-expression modules in rice and wheat. (**A**) Hierarchical clustering dendrogram and gene counts per co-expression module in rice; (**B**) Hierarchical clustering dendrogram and gene counts per co-expression module in wheat; (**C**–**E**) Expression patterns of representative rice modules, including M2 (**C**), M6 (**D**) and M9 (**E**); (**F**–**H**) Expression patterns of representative wheat modules, including M4 (**F**), M2 (**G**) and M7 (**H**). Heatmaps show gene-level expression across stress conditions, and bar plots summarize the mean Z-scored expression of each module under each treatment.

To organize stress-informative marker genes into coordinated transcriptional programs, we constructed weighted gene co-expression networks separately for rice and wheat using WGCNA (Figure 2). The hierarchical clustering dendrograms and module size distributions showed that the rice and wheat marker genes were partitioned into distinct co-expression modules (Figure 2A,B). After excluding genes assigned to the grey module, the rice network resolved 10 co-expression modules, whereas the wheat network resolved 14 modules. Complete gene-to-module assignments are provided separately for rice and wheat in Supplementary Tables S5 and S6, respectively. Correlation analysis between module eigengenes and stress traits showed that only a subset of modules displayed prominent module–trait associations (Supplementary Figure S1). We therefore focused on representative modules with clear module–trait relationships, stress-biased expression patterns and interpretable functional enrichment.

In rice, modules M2, M6 and M9 captured three prominent stress-associated programs (Figure 2C–E). Rice M2 showed a positive association with cold stress and a corresponding cold-biased expression pattern, indicating a distinct cold-responsive module. Rice M6 was most strongly associated with salt stress and enriched for photosynthesis and energy-related processes (Supplementary Figure S2B), suggesting coordinated regulation of metabolic functions during salinity stress. Rice M9 showed preferential activation under heat and salt conditions and was enriched for translation and macromolecule biosynthetic processes (Supplementary Figure S2D), consistent with a biosynthetic program engaged under stress.

The wheat network displayed a distinct set of stress-linked modules (Figure 2F–H). Wheat M4 was strongly associated with cold stress and enriched for cellular response to oxidative stress and protein folding (Supplementary Figure S3B), indicating a prominent cold-protective program. Wheat M2 showed the clearest positive relationship with drought stress and was enriched for response to abscisic acid and lipid biosynthetic processes (Supplementary Figure S3A), consistent with drought-associated hormonal and metabolic adjustment. Wheat M7 was positively associated with heat stress and linked to leaf development and regulation of DNA-templated transcription (Supplementary Figure S3C), suggesting transcriptional and developmental reprogramming under heat stress.

Together, these analyses show that stress-informative marker genes are organized into species-specific co-expression modules. These modules provide the network-context layer used below to interpret machine-learning-prioritized candidates and to test whether homologous stress-associated genes occupy comparable or divergent co-expression neighborhoods in rice and wheat.

### 2.3. Machine Learning Prioritizes Stress-Associated Candidate Genes in Rice

Supervised machine learning was next applied to cluster-specific marker genes to identify compact transcriptional signatures associated with abiotic stress states in rice. Within this framework, marker genes define the transcriptional state space of stress responses, WGCNA organizes these features into co-expression modules, and supervised learning further prioritizes a compact subset of stress-associated candidate genes for downstream biological interpretation.

**Figure 3:**
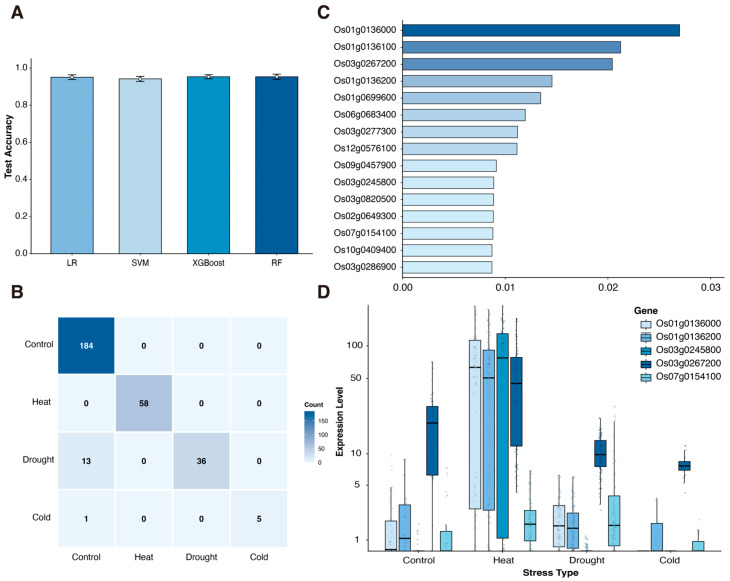
Machine-learning classification and prioritization of stress-associated candidate genes in rice. (**A**) Independent test accuracy of four machine-learning classifiers; (**B**) Confusion matrix of the Random Forest classifier on the independent test dataset, with rows representing true classes and columns representing predicted classes; (**C**) Random Forest feature-importance scores based on mean decrease in impurity for representative stress-associated candidate genes; (**D**) Expression distributions of representative rice candidate genes across stress conditions. RF, Random Forest; SVM, support vector machine; LR, logistic regression.

Using a subset of curated rice leaf RNA-seq projects selected from Supplementary Table S1, we optimized and evaluated four machine-learning classifiers. Random Forest achieved the highest independent test accuracy of 0.953 (Figure 3A). The confusion matrix showed limited misclassification among the major stress classes, indicating that the marker-gene feature set retained strong stress-state information after data harmonization (Figure 3B). Internal five-fold cross-validation during grid-search optimization showed that the relative performance patterns of the classifiers were generally consistent across validation folds (Supplementary Figure S4). Although cross-validation accuracies were lower than training accuracies, the high independent test accuracy suggested reasonable model generalization.

Having established that marker-gene expression effectively resolved stress-associated transcriptional states, we next examined Random Forest impurity-based feature-importance scores to prioritize genes contributing to the classification structure (Figure 3C). The complete list of RF-ranked rice candidate genes is provided in Supplementary Table S7. Among the highly ranked heat-associated candidates, *Os01g0136000*, annotated as *OsHSP16.9C*, and *Os01g0136200* emerged as prominent features. The thermal induction of *OsHSP16.9C* has been linked to heat-shock elements in its promoter, which are important for maintaining proteostasis under high-temperature stress [29]. For drought-related responses, the model prioritized *Os07g0154100*, encoding *OsNCED4*, a rate-limiting enzyme in ABA biosynthesis recently shown to be stabilized through *DRG9*-mediated recruitment into stress granules [30]. Additionally, *Os03g0286900*, annotated as *OsRCI2-5*, was retained as a stress-associated candidate; its reported membrane localization and drought-resistance phenotype are consistent with potential roles in stress signaling or membrane stability [31]. The model also identified *Os03g0245800* as a stress-associated candidate; this locus has been linked to salt-related tolerance traits in rice [32]. Expression distributions of representative candidates further supported their stress-biased behavior across the harmonized rice atlas (Figure 3D).

### 2.4. Machine Learning Prioritizes Stress-Associated Candidate Genes in Wheat

To assess whether the same machine-learning framework could resolve stress-associated transcriptional states in wheat, we applied the marker-gene-based classification strategy to a subset of curated wheat leaf RNA-seq projects selected from Supplementary Table S2. This analysis also provided an opportunity to evaluate candidate-gene prioritization in a polyploid cereal species with extensive homoeologous complexity.

**Figure 4:**
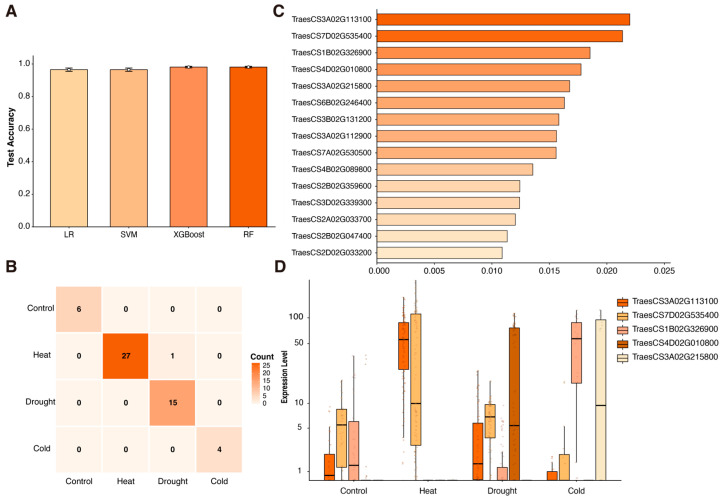
Machine-learning classification and prioritization of stress-associated candidate genes in wheat. (**A**) Independent test accuracy of four machine-learning classifiers; (**B**) Confusion matrix of the Random Forest classifier on the independent test dataset, with rows representing true classes and columns representing predicted classes; (**C**) Random Forest feature-importance scores based on mean decrease in impurity for representative stress-associated candidate genes; (**D**) Expression distributions of representative wheat candidate genes across stress conditions. RF, Random Forest; SVM, support vector machine; LR, logistic regression.

Hyperparameters for each classifier were optimized using grid search with stratified five-fold cross-validation on the training dataset. Random Forest achieved the highest independent test accuracy of 0.981 (Figure 4A), and its confusion matrix showed reliable separation among the four classes with limited misclassification (Figure 4B). Mean cross-validation accuracies and their variability across folds during hyperparameter optimization were generally consistent (Supplementary Figure S4). Similarly, although cross-validation accuracies were lower than training accuracies, the high independent test accuracy suggested reasonable model generalization.

We next examined impurity-based Random Forest feature-importance rankings to identify genes contributing most strongly to the wheat stress-associated classification structure (Figure 4C). The complete list of RF-ranked wheat candidate genes is provided in Supplementary Table S8. Several highly ranked candidates were associated with heat protection, including HSP20/α-crystallin-domain chaperone candidates such as *TraesCS3A02G113100* and *TraesCS3B02G131200*. The early light-induced protein candidate *TraesCS4D02G010800* was also retained as a heat-associated feature, consistent with previous wheat RNA-seq evidence showing transcriptional changes among wheat varieties with different heat-stress susceptibilities [33]. These features are consistent with the central importance of chaperone activity, protein folding and chloroplast-associated protection during thermal stress in wheat. Other highly ranked genes, including *TraesCS2B02G047400*, further suggest that the wheat model captured not only canonical heat-shock components, but also additional stress-associated transcriptional features.

Representative wheat candidates showed clear stress-biased expression distributions across the harmonized atlas (Figure 4D). Together, these results indicate that supervised learning can prioritize a compact set of biologically interpretable wheat stress-associated candidate genes from the integrated marker-gene feature layer. These RF-ranked candidates provided the wheat reference set for subsequent cross-species comparison with rice stress-associated genes.

### 2.5. Orthology-Guided Comparison Reveals Conserved Stress Responsiveness but Divergent Co-Expression Contexts

**Figure 5:**
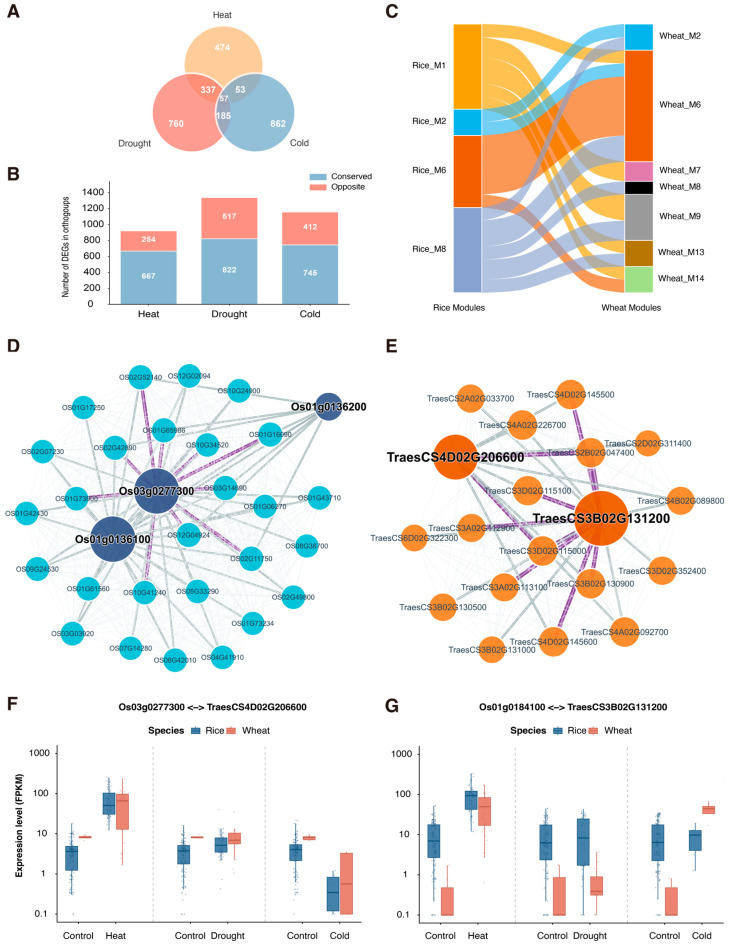
Orthology-guided comparison reveals conserved stress responsiveness and divergent co-expression contexts between rice and wheat. (**A**) Overlap of stress-responsive orthologous pairs across shared heat, drought and cold contrasts; (**B**) Numbers of orthologous pairs showing conserved or opposite expression directions across shared stress contrasts; (**C**) Correspondence between rice and wheat WGCNA modules based on shared orthologous genes; (**D**,**E**) Representative local co-expression subnetworks centered on ortholog-linked heat-shock/chaperone-related candidates in rice (**D**) and wheat (**E**); (**F**,**G**) Expression distributions of representative orthologous pairs across control and stress conditions, including the HSP70-family pair *Os03g0277300*/*OsHSP70*–*TraesCS4D02G206600* (**F**) and the small heat-shock/chaperone-related pair *Os01g0184100*/*OsHSP18.0-CII*/*OsHSP18.2*–*TraesCS3B02G131200*/HSP20/α-crystallin-domain candidate (**G**).

To assess whether stress-associated transcriptional responses were conserved between rice and wheat, we first compared high-confidence rice–wheat orthologous pairs across shared abiotic stress contrasts. The rice–wheat orthologous relationships used for this analysis are provided in Supplementary Table S9. Most stress-responsive orthologous pairs were condition-specific, with only a subset shared across heat, drought and cold contrasts (Figure 5A). Classification by expression direction further showed that some orthologous pairs retained concordant stress responsiveness, whereas others displayed opposite or stress-specific behavior between species (Figure 5B). Representative heatmaps illustrating cross-species stress-response patterns of orthologous pairs are shown in Supplementary Figure S5. These results indicate that homologous stress-responsive genes include a limited conserved component but also substantial stress-dependent divergence.

We next linked orthologous relationships to co-expression organization by connecting rice and wheat WGCNA modules through shared orthologous genes. Rather than forming a simple one-to-one correspondence, rice and wheat modules showed many-to-many associations, with individual modules in one species connected to multiple modules in the other (Figure 5C). This pattern suggests that conserved or homologous stress-associated genes are not necessarily embedded in equivalent co-expression neighborhoods but can be redistributed across species-specific module architectures.

To examine this co-expression context divergence at higher resolution, we focused on two representative heat-shock/chaperone-related orthologous pairs prioritized by the machine-learning framework. The first pair consisted of *Os03g0277300*, annotated as *OsHSP70*, and its wheat counterpart *TraesCS4D02G206600*, annotated as an HSP70-family protein. The second pair consisted of *Os01g0184100*, annotated as *OsHSP18.0-CII*/*OsHSP18.2*, and its wheat counterpart *TraesCS3B02G131200*, which contains an HSP20/α-crystallin chaperone domain. Local co-expression subnetworks showed that these paired genes were embedded in connected stress-associated neighborhoods in both species, but the surrounding co-expressed genes and local connectivity patterns differed between rice and wheat (Figure 5D,E). Expression profiling further indicated that the HSP70-family pair showed broadly concordant stress-associated behavior, whereas the small heat-shock/chaperone-related pair displayed stronger cross-species differences in expression magnitude and stress specificity (Figure 5F,G). Together, these analyses show that homologous heat-shock/chaperone-related candidates can retain stress-associated expression while being redeployed into divergent co-expression contexts.

### 2.6. Leaf Single-Cell Projection Reveals Divergent Cell-Type Deployment of Homologous Stress-Associated Candidates

**Figure 6:**
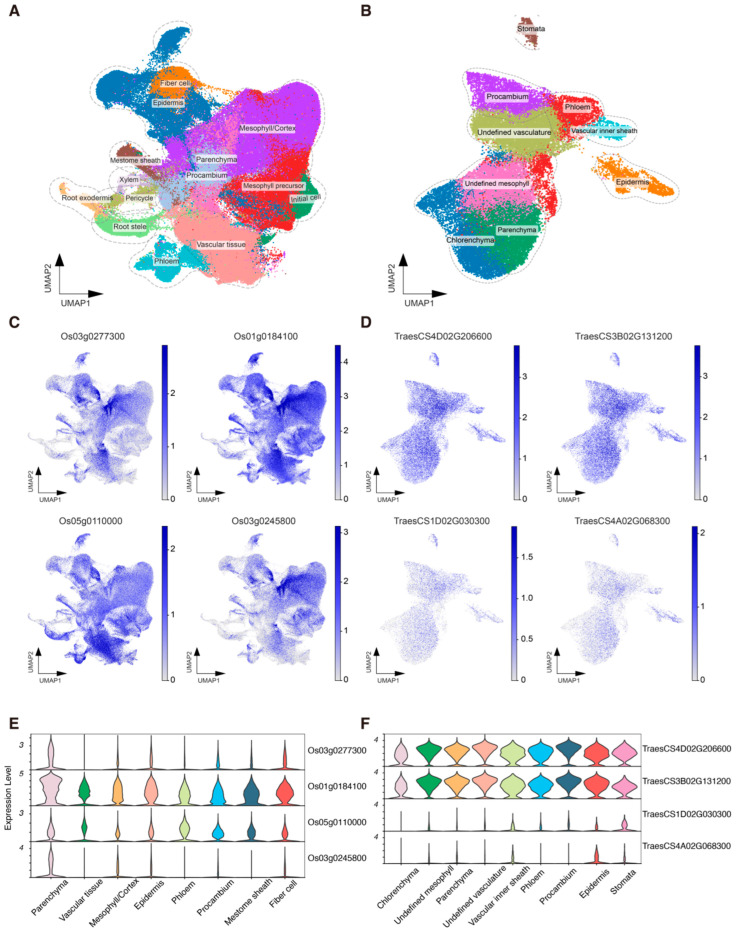
Leaf single-cell projection reveals divergent cellular deployment of homologous stress-associated candidates. (**A**,**B**) UMAP visualizations of rice (**A**) and wheat (**B**) leaf single-cell RNA-seq atlases, colored by annotated cell type; (**C**) Feature plots showing normalized expression of selected machine-learning-prioritized rice candidates, including *Os03g0277300*, *Os01g0184100*, *Os05g0110000* and *Os03g0245800*; (**D**) Feature plots showing normalized expression of matched wheat orthologs, including *TraesCS4D02G206600*, *TraesCS3B02G131200*, *TraesCS1D02G030300* and *TraesCS4A02G068300*; (**E**,**F**) Violin plots quantifying expression levels of the selected rice candidates (**E**) and wheat orthologs (**F**) across annotated leaf cell types.

To determine whether homologous stress-associated genes occupy comparable cellular contexts in rice and wheat leaves, we projected selected machine-learning-prioritized candidates onto species-specific leaf single-cell RNA-seq atlases [25,28]. This analysis focused on ortholog-linked candidates identified above, particularly the heat-shock/chaperone-related pairs used for co-expression comparison. The rice and wheat leaf atlases resolved major cellular compartments, allowing us to examine whether bulk-derived stress-associated genes were broadly expressed across leaf tissues or preferentially enriched in defined leaf cell types (Figure 6A,B).

In rice, several selected candidates exhibited cell-type-associated expression patterns. *Os03g0277300*, annotated as *OsHSP70*, was preferentially enriched in parenchyma-associated cells, indicating compartmentalized deployment of this HSP70-family heat-shock candidate within the rice leaf (Figure 6C,E). Similarly, *Os03g0245800* showed preferential expression in vascular-associated cell populations, suggesting that multiple rice stress-associated candidates were linked to defined cellular compartments. By contrast, *Os01g0184100*, annotated as *OsHSP18.0-CII*/*OsHSP18.2*, displayed a broader expression pattern across multiple rice leaf cell lineages. These observations indicate that cell-type enrichment was gene-dependent rather than a universal property of all heat-shock or stress-associated candidates.

The matched wheat orthologs were detected across a wider range of leaf cell populations. This cross-species contrast was most evident for the HSP70-family pair: *Os03g0277300*/*OsHSP70* was enriched in rice parenchyma-associated cells, whereas its wheat counterpart *TraesCS4D02G206600*, annotated as an HSP70-family protein, showed broader expression across multiple wheat leaf cell types, including mesophyll-, epidermal- and vascular-associated clusters (Figure 6D,F). *TraesCS3B02G131200*, the HSP20/α-crystallin-domain counterpart of *Os01g0184100*/*OsHSP18.0-CII*, was also broadly detected across wheat leaf cell types, consistent with a more distributed chaperone-related expression pattern in wheat. Quantitative violin plots supported the feature-plot observations, showing that selected rice candidates included more cell-type-enriched expression patterns, whereas their wheat counterparts were generally more broadly distributed across the leaf cellular landscape.

These results extend the orthology-guided co-expression analysis by showing that homologous stress-associated candidates can differ not only in co-expression neighborhood structure but also in leaf cell-type deployment. The HSP70-family pair provides a representative example of this cross-species divergence, linking parenchyma-enriched expression in rice with broader cellular deployment in wheat.

## 3. Discussion

Our study suggests that abiotic stress responses in rice and wheat are shaped not only by the conservation of stress-associated genes, but also by the redeployment of homologous genes across species-specific co-expression and cellular contexts. By integrating public leaf transcriptomes, co-expression networks, supervised machine learning, orthology-guided comparison and single-cell transcriptomic projection, we found that rice and wheat share major stress-related molecular functions but organize them through distinct transcriptional architectures. Rice samples formed relatively discrete stress-associated transcriptional states, whereas wheat samples occupied a more continuous expression landscape with greater overlap among stress categories (Figure 1) [10]. Consistently, stress-informative genes were assembled into species-specific co-expression modules, with rice modules associated with cold response, photosynthesis-related processes and biosynthetic programs, while wheat modules highlighted oxidative stress protection, abscisic acid signaling and transcriptional regulation (Figure 2) [11,12,15,16]. These findings extend previous transcriptomic studies of cereal stress responses by showing that shared stress-associated pathways do not necessarily occupy equivalent network contexts across species.

This context-dependent divergence may partly reflect the contrasting genome architectures of rice and wheat. Rice has a compact diploid genome, whereas bread wheat is a hexaploid allopolyploid characterized by extensive gene redundancy and homoeolog diversity. Previous studies have shown that wheat exhibits complex homoeolog expression and tissue-dependent transcriptional regulation [10]. In this context, the more discrete stress-associated states observed in rice may represent a more compartmentalized transcriptional strategy, whereas the broader wheat landscape may reflect regulatory buffering and transcriptional plasticity associated with polyploid genome organization [34]. Thus, wheat stress responses should not be viewed simply as stronger or weaker versions of rice responses, but rather as programs that may be distributed across a wider set of transcriptional and cellular compartments.

Machine-learning-based prioritization provided a focused entry point for identifying stress-associated candidate genes from these large transcriptomic resources. The evaluated classifiers captured substantial stress-state information from the marker-gene feature sets, and Random Forest was subsequently used for candidate-gene prioritization because it provides interpretable feature-importance estimates (Figure 3 and Figure 4) [21]. The top-ranked candidates included biologically interpretable genes related to heat-shock responses, ABA biosynthesis, membrane-associated stress responses and chloroplast-associated protection [35,36,37]. The recovery of known stress-related genes supports the utility of machine learning for candidate prioritization, but these candidates should be interpreted as informative predictors rather than functionally validated regulators.

Recent applications of machine learning in plant stress biology have demonstrated its value for predicting stress-responsive genes and identifying informative molecular features from transcriptomic datasets [20,21]. Previous ML studies have primarily focused on stress prediction or feature prioritization within individual species. Our framework extends these studies by integrating three complementary analytical layers within a comparative evolutionary context. WGCNA provides systems-level organization of transcriptional programs, supervised machine learning prioritizes compact sets of biologically interpretable stress-associated genes, and single-cell transcriptomic projection resolves their cellular deployment. Rather than treating prediction as the final objective, our framework uses machine learning as one component of a broader biological inference strategy, enabling candidate genes to be interpreted simultaneously through network topology, orthologous relationships and cell-type specificity. This multi-layered integration allows comparative transcriptomics to move beyond gene lists toward mechanistic hypotheses regarding regulatory conservation and divergence across cereal species.

A central implication of our analysis is that orthology alone is insufficient to infer conserved regulatory deployment. Orthology-based comparisons often assume that homologous genes provide a direct bridge between species, but our results indicate that conservation of gene identity does not necessarily imply conservation of co-expression organization or cellular expression distribution (Figure 5). Only a subset of stress-responsive orthologous pairs showed shared responses across heat, drought and cold conditions, whereas many others displayed condition-specific or divergent expression patterns. Moreover, rice and wheat co-expression modules showed many-to-many relationships through shared orthologous genes rather than simple one-to-one correspondence [38]. These observations support a model of co-expression rewiring, in which conserved stress-associated genes retain aspects of ancestral function while becoming incorporated into species-specific transcriptional programs [39,40].

The heat-shock/chaperone-related candidates provide a representative example of this redeployment. HSP70 and small heat-shock proteins are core components of plant proteostasis, but their cross-species behavior in this study was not limited to conserved stress responsiveness. The HSP70-family pair *Os03g0277300*–*TraesCS4D02G206600* and the small heat-shock protein pair *Os01g0184100*–*TraesCS3B02G131200* retained stress-associated expression, yet differed in their local co-expression neighborhoods and connectivity patterns [41,42]. Single-cell projection further suggested that this divergence extends to the cellular level. For example, *Os03g0277300* showed preferential expression in rice large parenchyma cells, whereas its wheat ortholog *TraesCS4D02G206600* was detected across broader leaf cell populations, including mesophyll-, epidermal- and vascular-associated clusters (Figure 6) [25,43]. These findings raise the possibility that conserved stress-protective machinery can be reorganized in a species-specific manner across both network and leaf cellular landscapes.

The divergent cellular deployment of orthologous stress-associated genes observed here is likely to reflect multiple evolutionary processes operating since the divergence of rice and wheat. Bread wheat has experienced polyploidization followed by extensive homoeolog retention, regulatory diversification and subfunctionalization, whereas rice retains a comparatively compact diploid genome. Such evolutionary trajectories can preserve core stress responsiveness while redistributing transcriptional activity among different regulatory modules and cell types. In addition, neofunctionalization of duplicated genes, cis-regulatory evolution and lineage-specific rewiring of transcription-factor networks may further contribute to differences in cellular expression domains despite conserved molecular functions. Consequently, homologous genes should not necessarily be expected to occupy equivalent transcriptional neighborhoods or cellular compartments across species. Instead, our findings support the view that evolutionary conservation primarily operates at the level of biological function, whereas regulatory implementation can remain remarkably flexible across different genomic architectures.

More broadly, these findings have implications for comparative crop genomics and candidate-gene prioritization. Orthology-based approaches remain valuable for transferring biological knowledge between model species and crops, but our results suggest that homologous genes should be interpreted together with their stress-response patterns, co-expression neighborhoods and cell-type-associated expression. For climate-resilience breeding, the most informative candidates may therefore be genes that combine stress association, interpretable network context and relevant cellular expression patterns, rather than genes selected solely by expression magnitude or conservation status.

Despite these insights, several limitations should be considered. WGCNA captures co-expression rather than direct regulatory interactions, machine-learning feature importance prioritizes candidates but does not establish causality, and reciprocal best-hit orthology cannot fully resolve the complexity of wheat homoeologs [44]. In addition, single-cell projection provides cell-type-associated expression information rather than direct spatial localization or stress-induced single-cell dynamics [45]. Future work combining controlled cross-species stress time courses, homoeolog-resolved analyses, spatial transcriptomics and functional perturbation will be needed to test whether the candidates identified here directly contribute to stress tolerance. An additional consideration is the use of heterogeneous public RNA-seq datasets. Although batch effects were minimized using ComBat while preserving biological treatment effects, residual variation associated with experimental design, developmental stage, genotype, tissue sampling, sequencing depth and laboratory-specific protocols cannot be completely eliminated. These latent confounding factors may influence network topology, feature-importance estimates and comparative expression patterns. Nevertheless, integrating hundreds of independently generated transcriptomes also increases biological breadth and enables the identification of stress-associated signatures that are consistently recovered across multiple experimental backgrounds. Future studies based on standardized cross-species experimental designs and matched stress treatments will provide an important opportunity to validate and refine the comparative patterns identified here.

Overall, our study demonstrates that conserved abiotic stress genes cannot be fully understood through sequence conservation or differential expression alone. Instead, integrating co-expression topology, machine learning-guided candidate prioritization and single-cell transcriptomic context provides an additional layer of biological interpretation in which conserved stress-associated genes are redeployed across distinct transcriptional neighborhoods and cellular compartments. By combining comparative transcriptomics across a diploid and a hexaploid cereal with WGCNA, supervised machine learning and single-cell projection, our framework provides a scalable strategy for dissecting the evolutionary rewiring of stress programs and for identifying biologically interpretable candidate genes for future functional validation and climate-resilient crop improvement.

## 4. Materials and Methods

### 4.1. RNA-Seq Data Collection and Preprocessing

Raw leaf RNA-seq datasets for *Oryza sativa* and *Triticum aestivum* were retrieved from the NCBI Sequence Read Archive (SRA). Accession numbers, project identifiers, sample metadata, treatment annotations and source publications are provided separately for rice and wheat in Supplementary Tables S1 and S2, respectively. Rice and wheat reads were processed against the *Oryza sativa* ssp. *japonica* Nipponbare IRGSP-1.0 reference genome [8] and the IWGSC RefSeq wheat reference genome [9], respectively.

Datasets were retained when they were generated from leaf tissue, contained clearly annotated control and abiotic-stress treatments, included matched control and stress-treated samples with at least two biological replicates per treatment group, and provided publicly accessible raw sequencing reads. Samples with ambiguous treatment annotations, incomplete metadata, unsuitable tissue sources, or insufficient biological replication were excluded.

Raw sequencing data were downloaded using iSeq v1.9.8 [46], and read processing and quantification were performed through the iOmics pipeline. Briefly, low-quality bases and adapter sequences were removed using fastp v1.3.3 [47], and the cleaned reads were aligned to the corresponding reference genome using STAR v2.7.11b [48]. Alignment files were processed and indexed using sambamba v1.0.1 [49]. Gene- and isoform-level expression values were quantified using RSEM v1.3.3 [50]. Gene-level FPKM values were retained as the primary expression measure for downstream normalization, batch correction, transcriptomic atlas construction, co-expression network analysis and machine-learning classification. When genome-browser visualization was required, CPM-normalized bigWig files were generated using deepTools v3.5.6 [51].

### 4.2. Data Normalization and Batch-Effect Correction

Gene-level expression values were transformed using log_2_(FPKM + 1) before downstream analysis. To reduce the influence of low-abundance transcripts, genes with FPKM > 1 in at least 10% of samples within each species were retained for atlas construction and downstream analyses. Rice and wheat datasets were processed separately throughout normalization and batch-effect correction.

To reduce technical variation across independent studies, batch-effect correction was performed separately for rice and wheat in R v4.5.1 using the ComBat function in the sva package v3.60.0 [52,53]. Project or study accession was specified as the batch variable. To preserve biologically relevant stress-associated variation, stress condition or treatment group was included as a biological covariate in the ComBat model matrix where applicable. ComBat was run using the default parametric empirical Bayes settings.

The resulting harmonized expression matrices were used for dimensionality reduction, marker-gene identification, co-expression network analysis and machine-learning classification. The effectiveness of batch-effect correction was evaluated by comparing sample distributions before and after correction using PCA or UMAP projections colored by project accession and stress condition.

### 4.3. Construction of Integrated Stress Atlases and Identification of Cluster-Specific Marker Genes

Integrated stress atlases were constructed separately for rice and wheat using Seurat v5.5.1 [54]. For each species, normalized and batch-corrected leaf expression matrices were used as input, with bulk RNA-seq samples treated as observations. Principal component analysis was first performed to reduce dimensionality, followed by UMAP visualization to represent major axes of stress-associated transcriptional variation [55]. UMAP embeddings were generated using the first 30 principal components for rice and the first 20 principal components for wheat. For wheat, UMAP was run with n.neighbors = 15 and min.dist = 0.3; other parameters followed Seurat defaults unless otherwise specified.

Graph-based clustering was performed using the Louvain community detection algorithm [56] implemented in Seurat. Clustering was performed at a resolution of 0.5 for rice and 0.6 for wheat, yielding ten clusters in rice and nine clusters in wheat. Cluster-specific marker genes were identified using Seurat differential expression analysis. Within each cluster, genes were ranked by average log_2_ fold change relative to all other clusters, and up to the 500 top-ranked genes were retained to generate a balanced marker-feature set across clusters. Marker statistics, including nominal *p*-values, average log_2_ fold change, detection fractions and adjusted *p*-values, are provided separately for rice and wheat in Supplementary Tables S3 and S4, respectively.

This procedure identified 3457 cluster-specific marker genes in rice and 4171 in wheat. These marker-gene sets were used as the stress-informative feature layer for downstream WGCNA and machine-learning analyses.

### 4.4. Co-Expression Network Analysis Using WGCNA

Weighted gene co-expression network analysis was performed separately for rice and wheat using the cluster-specific marker genes identified from the integrated stress atlases. Networks were constructed using the WGCNA R package v1.74 [19]. For each species, pairwise Pearson correlations were calculated across samples, and an unsigned topological overlap matrix was constructed. A soft-thresholding power of 6 was used for both rice and wheat to generate weighted co-expression networks.

Gene modules were detected by hierarchical clustering followed by dynamic tree cutting, with a minimum module size of 50 genes. Closely related modules were merged using species-specific eigengene dissimilarity thresholds, with mergeCutHeight = 0.25 for rice and mergeCutHeight = 0.35 for wheat. Genes assigned to the grey module were retained in the WGCNA output but were not assigned formal module numbers and were excluded from downstream biological interpretation.

To identify stress-associated modules, module eigengenes were correlated with treatment traits using Pearson correlation. Stress traits were encoded as one-hot binary variables, with each treatment represented as a separate trait. Representative modules discussed in the main text were selected based on module–trait associations, stress-biased expression patterns and functional interpretability. Gene-module assignments are provided separately for rice and wheat in Supplementary Tables S5 and S6, respectively.

### 4.5. Machine-Learning Models for Stress Classification and Candidate Prioritization

Supervised machine-learning models were trained separately for rice and wheat using cluster-specific marker-gene expression values as input features. Samples were divided into training and independent test sets using a stratified 70:30 split to preserve the relative distribution of stress classes. The multiclass classification task included four stress-response categories: Control, Heat, Drought and Cold.

Machine-learning analyses were performed in Python v3.11 using scikit-learn v1.9.0 [57] and XGBoost v3.3.0 [58]. Four classifiers with complementary learning strategies were evaluated: Random Forest [59], support vector machine (SVM), logistic regression and XGBoost. Random Forest and XGBoost were selected because of their ability to capture nonlinear relationships and complex feature interactions, whereas SVM and logistic regression were included as classical high-dimensional classification approaches and baseline models.

Marker-gene expression values were standardized using StandardScaler before fitting the SVM and logistic regression models. Class imbalance was addressed using balanced class weights where applicable.

Hyperparameter optimization was performed separately for each classifier using GridSearchCV with stratified five-fold cross-validation on the training dataset. The principal parameters evaluated included the number of trees, maximum tree depth and number of features considered at each split for Random Forest; kernel type, regularization parameter (*C*) and kernel coefficient (γ) for SVM; regularization penalty, inverse regularization strength (*C*) and solver for logistic regression; and the number of boosting trees, learning rate, maximum tree depth, minimum child weight, subsampling ratio and feature-sampling ratio for XGBoost. Candidate parameter combinations were ranked according to their mean cross-validation accuracy, and the combination achieving the highest mean accuracy was selected for each classifier. The complete parameter search spaces and selected optimal parameters are provided in Supplementary Table S10.

The optimized models were refitted using the complete training dataset and subsequently evaluated on the independent test dataset. Independent test accuracy was used to compare the four classifier types, while confusion matrices were used to examine class-specific prediction patterns. Mean cross-validation accuracy and its variability across the five folds during hyperparameter optimization are shown in Supplementary Figure S4.

Random Forest was used for candidate-gene prioritization because it provides an intrinsic and interpretable measure of feature importance. Feature importance was quantified as the mean decrease in impurity using the feature_importances_ attribute in scikit-learn. For each gene, the importance score reflects its average contribution to reducing node impurity across all trees in the forest. Genes with higher scores were prioritized as stronger predictive features for stress-state classification. These scores were used solely for candidate ranking and were not interpreted as evidence of direct or causal regulatory relationships.

### 4.6. Identification and Annotation of Rice–Wheat Orthologous Pairs

Putative orthologous relationships between rice and wheat were identified using a reciprocal best-hit BLASTN strategy based on coding sequences. Rice CDS sequences were obtained from the *Oryza sativa* ssp. *japonica* Nipponbare IRGSP-1.0 annotation in RAP-DB gene ID format [8], and wheat CDS sequences were obtained from the IWGSC RefSeq v6.1 annotation through Ensembl Plants [9]. For genes with multiple transcript isoforms, the longest CDS isoform was retained.

Reciprocal BLASTN searches were performed between rice and wheat CDS sequences using BLAST+ v2.16.0 [60]. BLASTN was run with max_target_seqs = 5, and hits were retained using an *E*-value cutoff of 10^−10^, minimum nucleotide identity of 70% and minimum alignment coverage of 50%. For each gene, the best hit in the opposite species was selected after filtering, and gene pairs recovered as mutual best hits were defined as high-confidence rice–wheat orthologous pairs. This procedure identified 16,381 reciprocal best-hit orthologous pairs.

Because bread wheat is a hexaploid allopolyploid with extensive homoeologous redundancy, these reciprocal best-hit pairs were treated as high-confidence one-to-one-like orthologous relationships rather than a complete catalog of all wheat homoeologs. Orthologous gene pairs are provided in Supplementary Table S9.

### 4.7. Classification of Stress-Responsive Orthologous Pairs

For cross-species comparison, rice–wheat reciprocal best-hit orthologous pairs with available expression values were compared across shared heat, drought and cold contrasts. For each species and stress contrast, stress responsiveness was defined using stress-versus-control log_2_ fold change calculated from normalized expression values. Genes with |log_2_FC| ≥ 1 were considered stress responsive.

Orthologous pairs were then classified according to the response status and expression direction of the paired rice and wheat genes. Pairs responsive in both species and changing in the same direction were classified as conserved, whereas pairs responsive in both species but changing in opposite directions were classified as opposite. Pairs responsive in only one species were classified as species-specific. The overlap analysis in Figure 5A was performed using orthologous pairs with available expression values in both species and at least one stress-responsive member under the corresponding contrast.

### 4.8. Representative Ortholog-Centered Subnetworks and Expression Comparison

Representative orthologous gene pairs were selected from machine-learning-prioritized candidates based on stress-associated expression, heat-shock/chaperone-related annotation, identifiable rice–wheat orthology and detectable expression in the corresponding leaf single-cell atlas. Representative pairs included *Os01g0184100*–*TraesCS3B02G131200* and *Os03g0277300*–*TraesCS4D02G206600*.

For each focal gene, local co-expression subnetworks were extracted from the species-specific WGCNA topological overlap matrix. To generate comparable local network representations, the focal gene and its strongest connected neighbors were retained for visualization. Because unsigned WGCNA networks were used, subnetworks were interpreted according to topological overlap strength rather than correlation direction.

Local co-expression subnetworks were compared visually between rice and wheat to assess divergence in co-expression context. No formal statistical test was applied to edge-weight distributions.

### 4.9. Single-Cell Atlas Projection and Cell-Type-Associated Expression Analysis

Public rice and wheat leaf single-cell RNA-seq atlases were used as cellular reference datasets. The processed rice leaf single-cell dataset, including normalized expression matrices, UMAP coordinates and cell-type annotations, was obtained from Wang et al. [25]. The processed wheat leaf single-cell dataset and corresponding annotations were obtained from Guo et al. [28].

Single-cell analyses were performed using Scanpy v1.12.2 [61]. For the rice atlas, the processed AnnData object provided by the original study (CRA004082) was used directly, and the original UMAP coordinates and annotated cell-type labels were retained. For the wheat atlas, expression matrices were normalized using total-count normalization followed by log transformation, after which PCA, Harmony-based batch correction, neighborhood graph construction, Leiden clustering and UMAP embedding were performed.

Candidate genes prioritized from bulk RNA-seq and machine-learning analyses were projected onto the corresponding species-specific leaf atlas. Wheat orthologs were matched to rice candidates using the orthology relationships described above. Cell-type-associated expression patterns were visualized using UMAP feature plots and violin plots. Expression distributions across annotated leaf cell types were compared to distinguish cell-type-enriched expression from broader cellular deployment.

### 4.10. Functional Enrichment Analysis

Functional enrichment analysis was performed for selected co-expression modules using Gene Ontology annotations [62]. Enrichment analysis was conducted using the clusterProfiler R package v4.20.0 [63] with species-specific gene-to-GO annotation tables. For each species, genes included in the corresponding WGCNA analysis were used as the background gene universe. Gene Ontology biological process terms were tested for over-representation. *p*-values were adjusted for multiple testing using the Benjamini–Hochberg method [64], and terms with an adjusted *p*-values below 0.05 were considered significant. Enrichment results were used to support the functional interpretation of representative stress-associated modules.

## 5. Conclusions

This study presents an integrative comparative framework for investigating abiotic stress-associated transcriptional programs in rice and wheat. By combining transcriptome-scale profiling, co-expression analysis, machine-learning-based candidate prioritization, orthology inference and single-cell transcriptomic projection, we examined stress-responsive genes within both network and cell-type-associated contexts, rather than relying solely on differential expression or sequence conservation.

Our analyses indicate that homologous stress-associated genes can retain stress responsiveness while occupying distinct co-expression neighborhoods and cellular expression patterns in different cereal species. These findings suggest that conservation of gene identity does not necessarily imply conservation of transcriptional organization. Instead, cereal stress adaptation may involve the redeployment of conserved molecular components into species-specific network and cellular contexts.

This integrative strategy provides a practical approach for prioritizing biologically interpretable candidates from large public transcriptomic datasets and for generating testable hypotheses regarding stress adaptation mechanisms. As transcriptomic and single-cell resources continue to expand, comparative analyses that incorporate multiple layers of biological information will be valuable for understanding how conserved genes acquire species-specific roles and for identifying candidate genes relevant to stress resilience and crop improvement in cereals.

## Data Availability

The original contributions presented in this study are included in the article/Supplementary Materials. Further inquiries can be directed to the corresponding author.

## Supplementary Materials

The following supporting information can be downloaded at: https://www.mdpi.com/article/10.3390/ijms27157005/s1.

## Abbreviations

The following abbreviations are used in this manuscript:

RNA-seq	RNA sequencing
SRA	Sequence Read Archive
NCBI	National Center for Biotechnology Information
ML	Machine learning
UMAP	Uniform Manifold Approximation and Projection
WGCNA	Weighted gene co-expression network analysis
GO	Gene Ontology
HSP	Heat-shock protein
ABA	Abscisic acid
FPKM	Fragments per kilobase of transcript per million mapped reads
PCA	Principal component analysis
CDS	Coding sequence

## References

[1] Shiferaw B., Smale M., Braun H.-J., Duveiller E., Reynolds M., Muricho G. (2013). Crops That Feed the World 10. Past Successes and Future Challenges to the Role Played by Wheat in Global Food Security. Food Secur..

[2] Seck P.A., Diagne A., Mohanty S., Wopereis M.C.S. (2012). Crops That Feed the World 7: Rice. Food Secur..

[3] Ray D.K., West P.C., Clark M., Gerber J.S., Prishchepov A.V., Chatterjee S. (2019). Climate Change Has Likely Already Affected Global Food Production. PLoS ONE.

[4] Zhao C., Liu B., Piao S., Wang X., Lobell D.B., Huang Y., Huang M., Yao Y., Bassu S., Ciais P. (2017). Temperature Increase Reduces Global Yields of Major Crops in Four Independent Estimates. Proc. Natl. Acad. Sci. USA.

[5] Lesk C., Rowhani P., Ramankutty N. (2016). Influence of Extreme Weather Disasters on Global Crop Production. Nature.

[6] Gupta A., Rico-Medina A., Caño-Delgado A.I. (2020). The Physiology of Plant Responses to Drought. Science.

[7] Zhang H., Zhu J., Gong Z., Zhu J.-K. (2022). Abiotic Stress Responses in Plants. Nat. Rev. Genet..

[8] Kawahara Y., de la Bastide M., Hamilton J.P., Kanamori H., McCombie W.R., Ouyang S., Schwartz D.C., Tanaka T., Wu J., Zhou S. (2013). Improvement of the *Oryza Sativa* Nipponbare Reference Genome Using next Generation Sequence and Optical Map Data. Rice.

[9] Appels R., Eversole K., Stein N., Feuillet C., Keller B., Rogers J., Pozniak C.J., Choulet F., Distelfeld A., The International Wheat Genome Sequencing Consortium (IWGSC) (2018). Shifting the Limits in Wheat Research and Breeding Using a Fully Annotated Reference Genome. Science.

[10] Ramírez-González R.H., Borrill P., Lang D., Harrington S.A., Brinton J., Venturini L., Davey M., Jacobs J., van Ex F., Pasha A. (2018). The Transcriptional Landscape of Polyploid Wheat. Science.

[11] Wilkins O., Hafemeister C., Plessis A., Holloway-Phillips M.-M., Pham G.M., Nicotra A.B., Gregorio G.B., Jagadish S.V.K., Septiningsih E.M., Bonneau R. (2016). EGRINs (Environmental Gene Regulatory Influence Networks) in Rice That Function in the Response to Water Deficit, High Temperature, and Agricultural Environments. Plant Cell.

[12] Cohen S.P., Leach J.E. (2019). Abiotic and Biotic Stresses Induce a Core Transcriptome Response in Rice. Sci. Rep..

[13] Derakhshani B., Lee C., Shin D., Jung K.-H. (2024). Identification of Core Drought-Responsive Genes for Developing Drought-Tolerant Rice Varieties through Meta-Analysis of RNA-Seq Data. Plant Biotechnol. Rep..

[14] Ahmdikhah A., Safaeizadeh M., Tehranian A.S. (2025). Responses of Rice Plant to Multiple Abiotic Stresses Revealed by Transcriptome Meta-Analysis and Identification of Novel Genetic Factors. Sci. Rep..

[15] Da Ros L., Bollina V., Soolanayakanahally R., Pahari S., Elferjani R., Kulkarni M., Vaid N., Risseuw E., Cram D., Pasha A. (2023). Multi-omics Atlas of Combinatorial Abiotic Stress Responses in Wheat. Plant J..

[16] Ding M., Fiaz S., Wan K., Chang H., Pan R., Admas T. (2025). Transcriptomic Meta-Analysis Reveals Hub Genes Integrating Multiple Abiotic Stress Responses in Wheat. Food Sci. Nutr..

[17] Zhou Y., Wang D., Wang H., Qiao Y., Zhao P., Cao Y., Liu X., Yang Y., Lin X., Xu S. (2025). Integrative Omics of the Genetic Basis for Wheat WUE and Drought Resilience Reveal the Function of *TaMYB7-A1*. Nat. Commun..

[18] Libbrecht M.W., Noble W.S. (2015). Machine Learning Applications in Genetics and Genomics. Nat. Rev. Genet..

[19] Langfelder P., Horvath S. (2008). WGCNA: An R Package for Weighted Correlation Network Analysis. BMC Bioinform..

[20] Gupta C., Ramegowda V., Basu S., Pereira A. (2021). Using Network-Based Machine Learning to Predict Transcription Factors Involved in Drought Resistance. Front. Genet..

[21] Smet D., Opdebeeck H., Vandepoele K. (2023). Predicting Transcriptional Responses to Heat and Drought Stress from Genomic Features Using a Machine Learning Approach in Rice. Front. Plant Sci..

[22] Sanchez-Munoz R., Depaepe T., Samalova M., Hejatko J., Zaplana I., Van Der Straeten D. (2025). Machine-Learning Meta-Analysis Reveals Ethylene as a Central Component of the Molecular Core in Abiotic Stress Responses in Arabidopsis. Nat. Commun..

[23] Panahi B., Hamid R., Mohammad Zadeh Jalaly H. (2025). Deciphering Plant Transcriptomes: Leveraging Machine Learning for Deeper Insights. Curr. Plant Biol..

[24] Tenorio Berrío R., Dubois M. (2024). Single-Cell Transcriptomics Reveals Heterogeneity in Plant Responses to the Environment: A Focus on Biotic and Abiotic Interactions. J. Exp. Bot..

[25] Wang Y., Huan Q., Li K., Qian W. (2021). Single-Cell Transcriptome Atlas of the Leaf and Root of Rice Seedlings. J. Genet. Genom..

[26] Liu Q., Liang Z., Feng D., Jiang S., Wang Y., Du Z., Li R., Hu G., Zhang P., Ma Y. (2021). Transcriptional Landscape of Rice Roots at the Single-Cell Resolution. Mol. Plant.

[27] Wang X., Huang H., Jiang S., Kang J., Li D., Wang K., Xie S., Tong C., Liu C., Hu G. (2025). A Single-Cell Multi-Omics Atlas of Rice. Nature.

[28] Guo X., Zhao A., Han J., Yuping L., Chen X., Cheng Z., Hou L., Lv L. (2025). Single-Cell Transcriptome Reveals the Cellular Response to PEG-Induced Stress in Wheat Leaves. J. Agric. Food Chem..

[29] Zhang Y., Zou B., Lu S., Ding Y., Liu H., Hua J. (2016). Expression and Promoter Analysis of the *OsHSP16.9C* Gene in Rice. Biochem. Biophys. Res. Commun..

[30] Wang H., Ye T., Guo Z., Yao Y., Tu H., Wang P., Zhang Y., Wang Y., Li X., Li B. (2024). A Double-Stranded RNA Binding Protein Enhances Drought Resistance via Protein Phase Separation in Rice. Nat. Commun..

[31] Li L., Li N., Song S.F., Li Y.X., Xia X.J., Fu X.Q., Chen G.H., Deng H.F. (2014). Cloning and Characterization of the Drought-Resistance *OsRCI2-5* Gene in Rice (*Oryza sativa* L.). Genet. Mol. Res..

[32] Li C., Lu C., Zou B., Yang M., Wu G., Wang P., Cheng Q., Wang Y., Zhong Q., Huang S. (2022). Genome-Wide Association Study Reveals a Genetic Mechanism of Salt Tolerance Germinability in Rice (*Oryza sativa* L.). Front. Plant Sci..

[33] Lee M.H., Kim K.-M., Sang W.-G., Kang C.-S., Choi C. (2022). Comparison of Gene Expression Changes in Three Wheat Varieties with Different Susceptibilities to Heat Stress Using RNA-Seq Analysis. Int. J. Mol. Sci..

[34] De Smet R., Van de Peer Y. (2012). Redundancy and Rewiring of Genetic Networks Following Genome-Wide Duplication Events. Curr. Opin. Plant Biol..

[35] Waters E.R. (2013). The Evolution, Function, Structure, and Expression of the Plant sHSPs. J. Exp. Bot..

[36] Xiang Z., Zhang L., Long Y., Zhang M., Yao Y., Deng H., Quan C., Lu M., Cui B., Wang D. (2024). An ABA Biosynthesis Enzyme Gene *OsNCED4* Regulates NaCl and Cold Stress Tolerance in Rice. Sci. Rep..

[37] Muthusamy S.K., Dalal M., Chinnusamy V., Bansal K.C. (2017). Genome-Wide Identification and Analysis of Biotic and Abiotic Stress Regulation of Small Heat Shock Protein (HSP20) Family Genes in Bread Wheat. J. Plant Physiol..

[38] Mutwil M., Klie S., Tohge T., Giorgi F.M., Wilkins O., Campbell M.M., Fernie A.R., Usadel B., Nikoloski Z., Persson S. (2011). PlaNet: Combined Sequence and Expression Comparisons across Plant Networks Derived from Seven Species. Plant Cell.

[39] Ovens K., Eames B.F., McQuillan I. (2021). Comparative Analyses of Gene Co-Expression Networks: Implementations and Applications in the Study of Evolution. Front. Genet..

[40] Alabdullah A.K., Borrill P., Martin A.C., Ramirez-Gonzalez R.H., Hassani-Pak K., Uauy C., Shaw P., Moore G. (2019). A Co-Expression Network in Hexaploid Wheat Reveals Mostly Balanced Expression and Lack of Significant Gene Loss of Homeologous Meiotic Genes Upon Polyploidization. Front. Plant Sci..

[41] Zhou Y., Lv W., Zhu M., Wang C., Yu L., Bao Y., Tang C., Zhang M., Huang Q., Ye D. (2025). Genome-Wide Analysis of the Hsp70 Gene Family in Rice Reveals That *OsHsp70-9* Plays a Significant Role in Heat Stress Response. BMC Plant Biol..

[42] Kumar A., Sharma S., Chunduri V., Kaur A., Kaur S., Malhotra N., Kumar A., Kapoor P., Kumari A., Kaur J. (2020). Genome-Wide Identification and Characterization of Heat Shock Protein Family Reveals Role in Development and Stress Conditions in *Triticum aestivum* L. Sci. Rep..

[43] Qiao L., Liu H., Li T., Ma Y., Ning J., Chang Z., Li J., Liu X., Zhao Z., Zheng X. (2026). Single-Nucleus RNA Sequencing Reveals Transcriptional Response of Wheat Roots and Leaves to Salt Stress. Crop J..

[44] Sharma N., Madan B., Khan M.S., Sandhu K.S., Raghuram N. (2023). Weighted Gene Co-Expression Network Analysis of Nitrogen (N)-Responsive Genes and the Putative Role of G-Quadruplexes in N Use Efficiency (NUE) in Rice. Front. Plant Sci..

[45] Johansen N., Hu H., Quon G. (2023). Projecting RNA Measurements onto Single Cell Atlases to Extract Cell Type-Specific Expression Profiles Using scProjection. Nat. Commun..

[46] Chao H., Li Z., Chen D., Chen M. (2024). iSeq: An Integrated Tool to Fetch Public Sequencing Data. Bioinformatics.

[47] Chen S. (2023). Ultrafast One-pass FASTQ Data Preprocessing, Quality Control, and Deduplication Using Fastp. iMeta.

[48] Dobin A., Davis C.A., Schlesinger F., Drenkow J., Zaleski C., Jha S., Batut P., Chaisson M., Gingeras T.R. (2013). STAR: Ultrafast Universal RNA-Seq Aligner. Bioinformatics.

[49] Tarasov A., Vilella A.J., Cuppen E., Nijman I.J., Prins P. (2015). Sambamba: Fast Processing of NGS Alignment Formats. Bioinformatics.

[50] Li B., Dewey C.N. (2011). RSEM: Accurate Transcript Quantification from RNA-Seq Data with or without a Reference Genome. BMC Bioinform..

[51] Ramírez F., Ryan D.P., Grüning B., Bhardwaj V., Kilpert F., Richter A.S., Heyne S., Dündar F., Manke T. (2016). deepTools2: A next Generation Web Server for Deep-Sequencing Data Analysis. Nucleic Acids Res..

[52] Leek J.T., Scharpf R.B., Bravo H.C., Simcha D., Langmead B., Johnson W.E., Geman D., Baggerly K., Irizarry R.A. (2010). Tackling the Widespread and Critical Impact of Batch Effects in High-Throughput Data. Nat. Rev. Genet..

[53] Johnson W.E., Li C., Rabinovic A. (2007). Adjusting Batch Effects in Microarray Expression Data Using Empirical Bayes Methods. Biostatistics.

[54] Stuart T., Butler A., Hoffman P., Hafemeister C., Papalexi E., Mauck W.M., Hao Y., Stoeckius M., Smibert P., Satija R. (2019). Comprehensive Integration of Single-Cell Data. Cell.

[55] McInnes L., Healy J., Melville J. (2018). Umap: Uniform manifold approximation and projection for dimension reduction. arXiv.

[56] Blondel V.D., Guillaume J.-L., Lambiotte R., Lefebvre E. (2008). Fast Unfolding of Communities in Large Networks. J. Stat. Mech. Theory Exp..

[57] Pedregosa F., Varoquaux G., Gramfort A., Michel V., Thirion B., Grisel O., Blondel M., Prettenhofer P., Weiss R., Dubourg V. (2011). Scikit-Learn: Machine Learning in Python. J. Mach. Learn. Res..

[58] Chen T., Guestrin C. (2016). XGBoost: A Scalable Tree Boosting System. Proceedings of the 22nd ACM SIGKDD International Conference on Knowledge Discovery and Data Mining, San Francisco, CA, USA, 13–17 August 2016.

[59] Breiman L. (2001). Random Forests. Mach. Learn..

[60] Camacho C., Coulouris G., Avagyan V., Ma N., Papadopoulos J., Bealer K., Madden T.L. (2009). BLAST+: Architecture and Applications. BMC Bioinform..

[61] Wolf F.A., Angerer P., Theis F.J. (2018). SCANPY: Large-Scale Single-Cell Gene Expression Data Analysis. Genome Biol..

[62] Carbon S., Douglass E., Good B.M., Unni D.R., Harris N.L., Mungall C.J., Basu S., Chisholm R.L., Dodson R.J., Hartline E. (2021). The Gene Ontology Consortium The Gene Ontology Resource: Enriching a GOld Mine. Nucleic Acids Res..

[63] Wu T., Hu E., Xu S., Chen M., Guo P., Dai Z., Feng T., Zhou L., Tang W., Zhan L. (2021). clusterProfiler 4.0: A Universal Enrichment Tool for Interpreting Omics Data. Innovation.

[64] Benjamini Y., Hochberg Y. (1995). Controlling the False Discovery Rate: A Practical and Powerful Approach to Multiple Testing. J. R. Stat. Soc. Ser. B Methodol..

