# Supplementary material for: Machine Learning-Guided Stress Atlases Reveal Co-Expression Rewiring and Divergent Cellular Deployment of Abiotic Stress Programs in Rice and Wheat
Source: Int J Mol Sci. 2026 Aug 4;27(15):7005. doi: 10.3390/ijms27157005 (PMC13466321; doi:10.3390/ijms27157005)
Supplement: Supplementary file 1 [file ijms-27-07005-s001.zip › ijms-4436647-supplementary/SupplementaryFiles/Supplementary Figures.pdf]

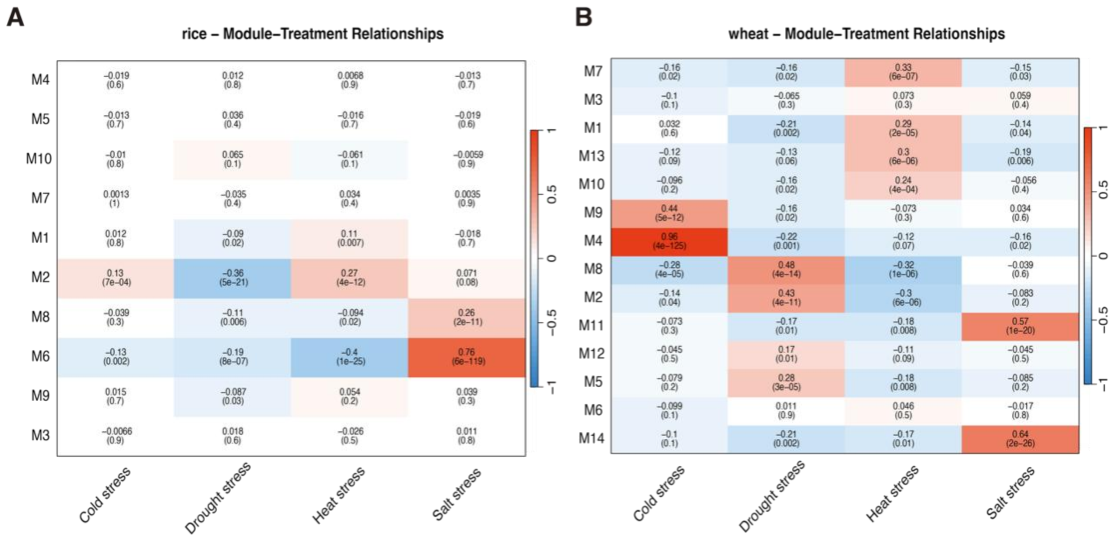

Figure S1. Module–trait relationships in rice and wheat. (A) Module–trait correlation heatmap for rice WGCNA modules. **(B)** Module–trait correlation heatmap for wheat WGCNA modules. Colors indicate Pearson correlation coefficients between module eigengenes and treatment traits. Numbers in each cell show correlation coefficients, with P values shown in parentheses.

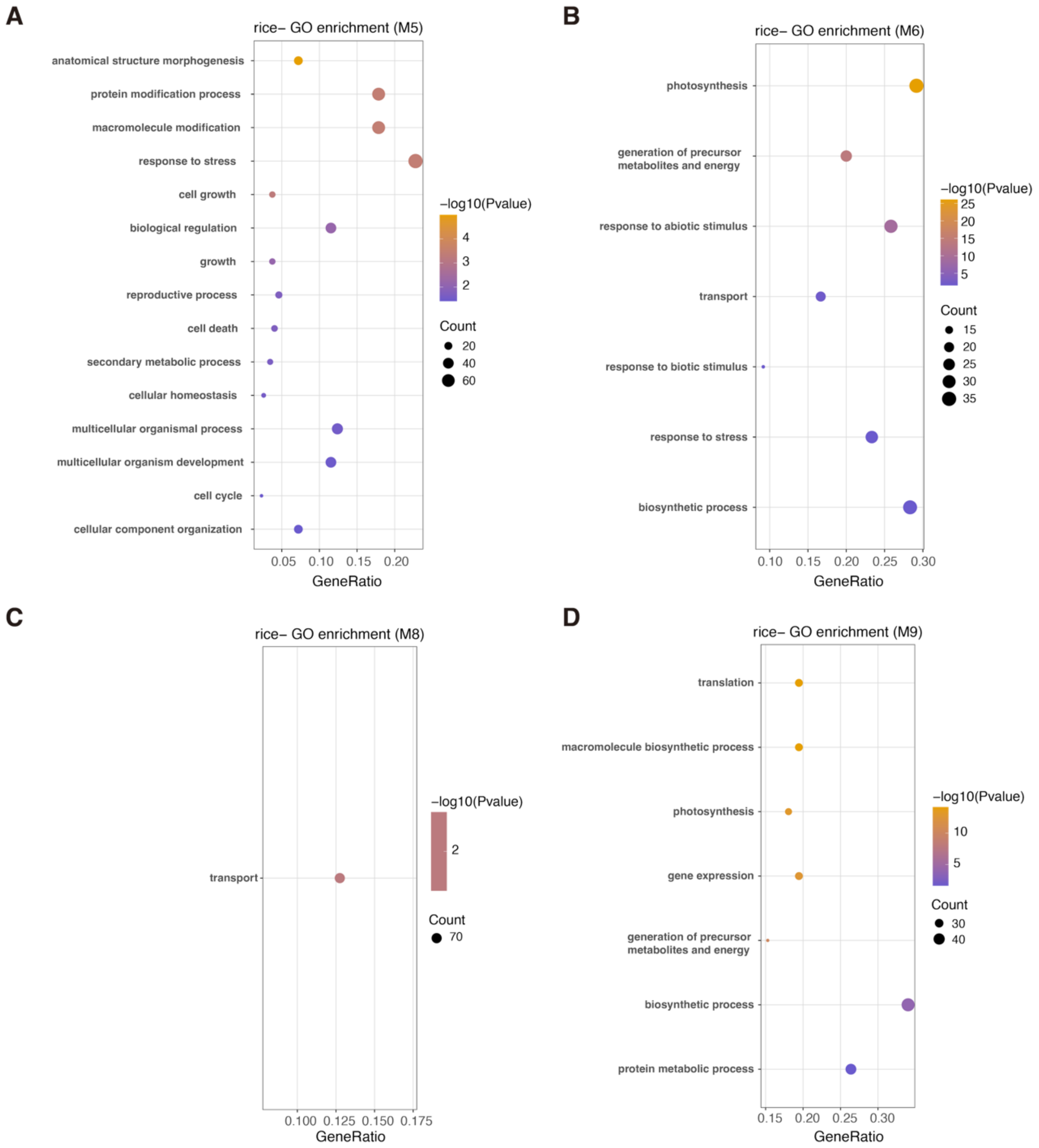

Figure S2. Functional enrichment of representative rice WGCNA modules. (A–D) Gene Ontology biological process enrichment results for representative rice modules M5 **(A)**, M6 **(B)**, M8 **(C)** and M9 **(D)**. Dot size indicates gene count, and color represents $-\log_{10}$(P value).

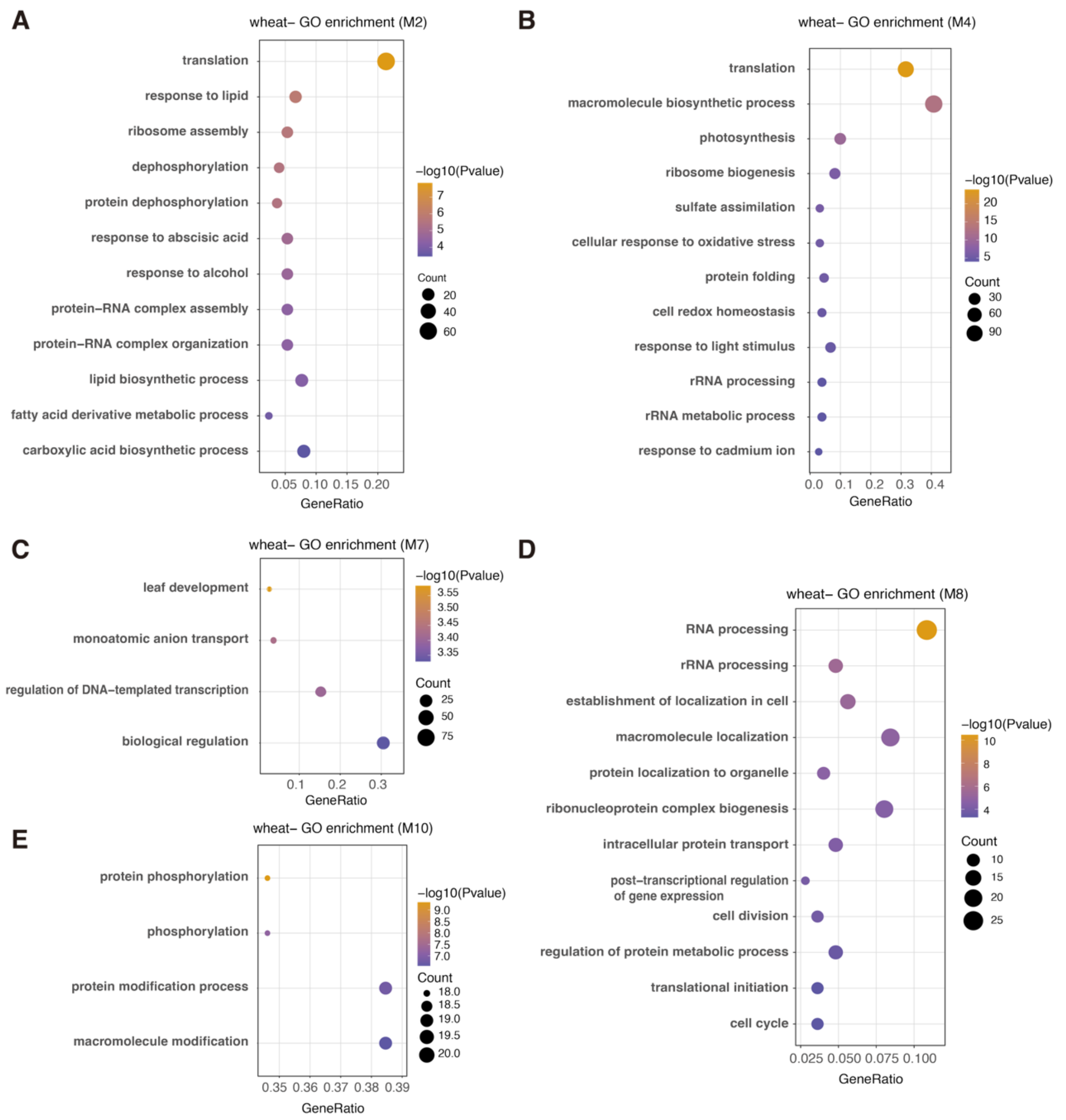

Figure S3. Functional enrichment of representative wheat WGCNA modules. (A–E) Gene Ontology biological process enrichment results for representative wheat modules M2 **(A)**, M4 **(B)**, M7 **(C)**, M8 **(D)** and M10 **(E)**. Dot size indicates gene count, and color represents $-\log_{10}$(P value).

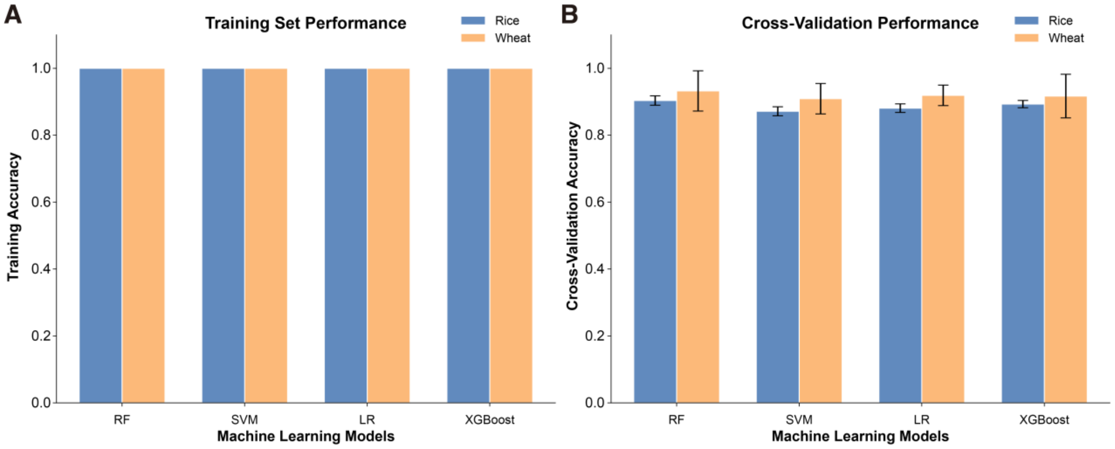

Figure S4. Training and five-fold cross-validation performance of the machine-learning classifiers. (A) Training accuracy of the optimized Random Forest, support vector machine, logistic regression and XGBoost classifiers in rice and wheat. **(B)** Mean accuracy obtained from stratified five-fold cross-validation during hyperparameter optimization. Error bars represent the standard deviation of accuracy across the five validation folds. Blue and orange bars represent rice and wheat, respectively. RF, Random Forest; SVM, support vector machine; LR, logistic regression; XGBoost, extreme gradient boosting.

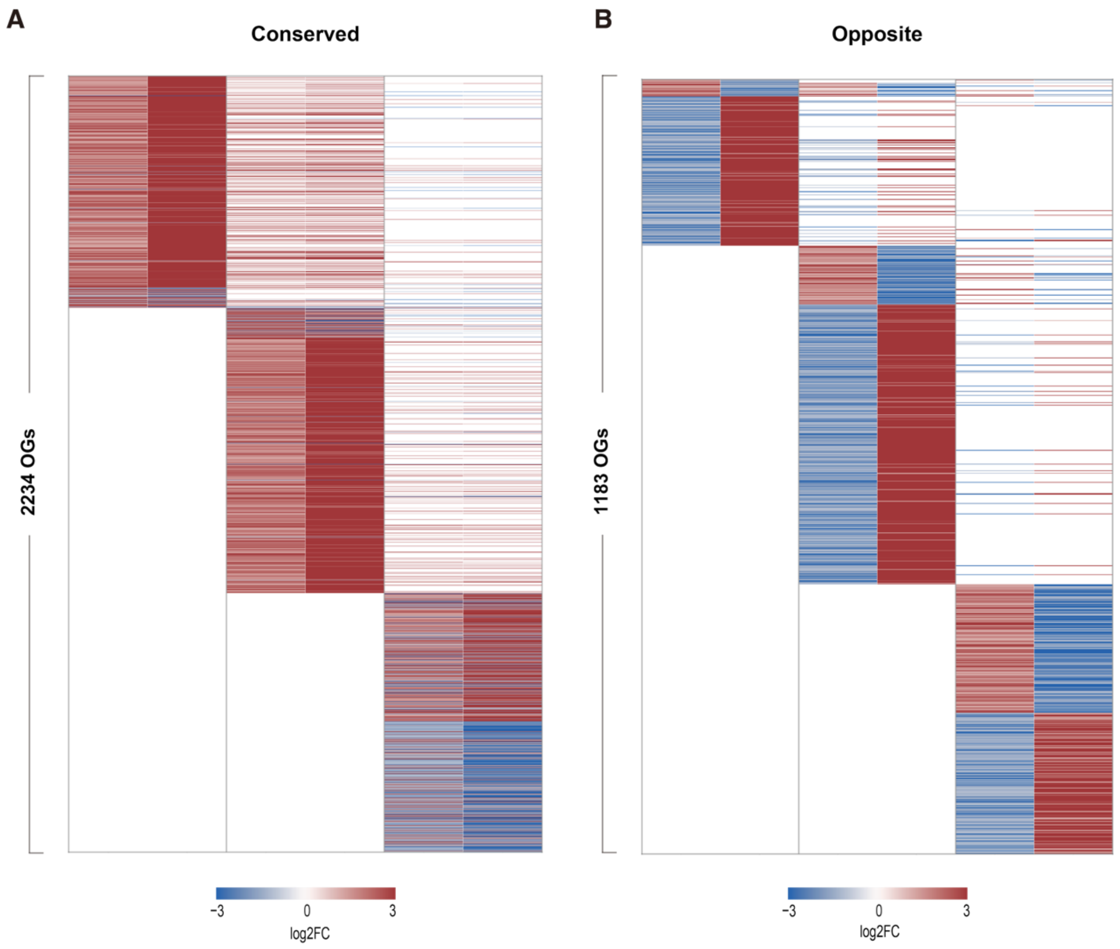

Figure S5. Cross-species expression patterns of representative rice–wheat orthologous pairs. (A) Heatmap of representative orthologous pairs with conserved stress-associated expression patterns. **(B)** Heatmap of representative orthologous pairs with opposite expression patterns. Colors indicate log2 fold change, with red and blue representing up- and down-regulation, respectively.
